# Simulation of Motor Core Gluing Process with Fine Mesh Nets

**DOI:** 10.3390/polym14214596

**Published:** 2022-10-29

**Authors:** Yong-Jie Zeng, Chia-Wei Liang, Sheng-Jye Hwang, Yu-Da Liu, Chien-Sheng Huang

**Affiliations:** 1 Department of Mechanical Engineering, National Cheng Kung University, Tainan 701401, Taiwan; 2 Department of Green Energy & System Integration Research, China Steel Corporation, Kaohsiung 812401, Taiwan; 3 Department of Metal Processing R&D, Metal Industries Research and Development Centre, Kaohsiung 811225, Taiwan

**Keywords:** iron core, fine mesh structure, computer-aided engineering tools, iron sheet, bonding

## Abstract

The actual process of using a resin to glue can optimize many shortcomings in the basic traditional process of welding a motor core. For example, the use of a resin for gluing can lead to a reduction in iron loss, improve rigidity, reduce processing times, and improve product quality. When using a gluing method, the biggest challenge is the distribution of the resin; therefore, resin distribution is very much important. This experiment used fine mesh nets to eventually improve the unbalanced state of resin distribution. In this research, in order to predict real flow behavior during gluing, computer-aided engineering was used for computer simulation. The results of the simulation showed that the illustrated trend of the filling process was very much similar to the actual experimental results. The simulation results could mostly predict defects and make effective improvements, which can lead to a significant reduction in the money and time spent on experiments. The simulation results of the dipping process also showed that the distribution of resin with fine mesh nets was more even than without fine mesh nets. Fine mesh nets can eventually improve an over-flow problem, which, ultimately, causes bumps. In this research, a simulation analysis of the gluing process of a motor core with fine mesh nets was conducted, and the results show that the resin distribution and the flow front of the runner were more even than those without fine mesh nets.

## 1. Introduction

In this research, the core result of resin distribution with fine mesh nets is entirely different from resin distribution without fine mesh nets. From previous research [1], resin distribution without fine mesh nets was always unbalanced, so fine mesh nets were added to the module during the filling process. From the results of the simulation analysis and the actual experiment, it was discovered that fine mesh nets can significantly improve the flow distribution of resin. From Seo’s [2] research, it was shown that the cutting and welding processes of a small stator core led to an increase in the residual stress, thereby resulting in increased iron losses. Therefore, the actual process of using a resin to glue in this research can help avoid the problem of increased iron losses. Scapin’s [3] research shows that the volume-of-fluid (VOF) method is reliable for mass conservation by construction. Zhou’s [4] research shows that the VOF model is effective at simulating the movement of a pressure surge and air pocket. From Chen’s [5] research, it was shown that a Castro–Macosko type of Equation can be used to describe a rise in isothermal viscosity as a function of the reaction conversion. Khalil Abdullah’s [6] research shows that the Castro–Macosko model is more reliable for the flow rheology of an epoxy molding compound (EMC), because it is consistent with the experimental results. It, essentially, includes the chemical kinetics of the cure reaction that actually determine the exact rate of conversion and its corresponding viscosity. From Chen’s [7] research, it was shown that Kamal’s model is reliable in cure kinetics and the Castro–Macosko model is considerably more stable and reliable in flow rheology. Moon’s [8] research shows that predicting viscosity by Kamal’s Equation is consistent with the viscosity measured by the rheometer. Azmi’s [9] research pointed out that the air trap, or a void that occurs at the chips, obviously, exacerbates flow retardation and also leads to a popcorn effect and least reliability. Aniszewski’s [10] research showed that the fluid volume method is always reliable in solving the free surface problem; its error is bounded, even on coarse grids. Lai’s [11] research shows that the mold filling simulation of Moldex3D showed a good correlation to the mold fronts, which was obtained by the process of short shots and actual void locations. Duan’s [12] research shows that the filling of different gate locations in the package, in fact, produces cavitation defects at different locations; a gate design with fewer corners in the injection direction also produces fewer cavitations. Sohail’s [13] research and Nazir’s [14,15] research show that acceleration slows down when porosity is increased. Therefore, a large number of pores in the fine meshes is considered in this research. In the case of resin flow, the free surface that occurs, when the fluid is in contact with the air, was actually calculated by the fluid volume method. In this research, in order to predict the flow trend of the resin and the distribution of the dipping process of different models, the methods of simulation analysis are proposed based on the previous research [1]. Based on all the above information, this study was well planned and simulated to investigate the flow front of the runner and the dipping process results.

## 2. Theory

### 2.1. Theoretical Background

The flow of resin in the runner is a three-dimensional transient behavior, and the mold flow analysis Equations used by the Moldex3D software to describe the non-isothermal resin flow in the runner were governing Equations, including continuity Equation, momentum Equation, and energy Equation. In the simulation, the governing Equation used the conservation principle of mass, momentum, and energy.

- Continuity Equation [16]

The continuity Equation represents the conservation of mass, which is used to ensure that the mass of the resin remains constant as it flows through the runner. The continuity Equation basically describes the mass conservation of filling with the resin:

(1) $$\frac{\partial \rho }{\partial t}+\nabla \cdot \left(\rho \vec{V}\right)=0$$

where $\vec{V}$ is the velocity vector; ρ is the density; and t  is the time.

- Momentum Equation [1]

The momentum Equation describes the fluid force changes during the filling process with the resin:

(2) $$\rho \left(\frac{\partial \vec{V}}{\partial t}+\vec{V}\cdot \nabla \vec{V}\right)=-\nabla P+\nabla \cdot \overset{\vec{⇀}}{\tau }+\rho \vec{g}$$

where $\vec{V}$ is the velocity vector; ρ is the density; t is the time; P is the pressure; $\vec{g}$ is the gravity; and $\overset{\vec{⇀}}{\tau }$ is the stress tensor.

- Energy Equation [17]

Based on the law of energy conservation, considering the heat and energy changes caused by heat conduction and the viscous dissipation of the resin during filling, the conservation of energy in the filling process is described by the actual energy Equation:

(3) $$\rho C_{P}\left(\frac{\partial T}{\partial t}+\vec{V}\cdot \nabla T\right)=\nabla \cdot \left(k\nabla T\right)+\eta {\dot{\gamma }}^{2}+\frac{d\alpha }{dt}\Delta H$$

where $\vec{V}$ is the velocity vector; ρ is the density; t is the time; *T* is the temperature; $C_{P}$ is the specific heat; α is the degree of cure; $\dot{\gamma }$ is the shear rate; ΔH is the reaction heat; and *η* is the viscosity taken as the generalized Cross–Castro–Macosko viscosity model [18]:

(4) $$\eta =\frac{\eta_{0}{\left(\frac{\alpha_{g}}{\alpha_{g}-\alpha }\right)}^{C_{1}+C_{2}\alpha }}{1+{\left(\frac{\eta_{0}\dot{\gamma }}{\tau^{*}}\right)}^{1-n^{*}}}$$

(5) $$\eta_{0}=A_{1}\cdot \exp\left(\frac{T_{b}}{T}\right)$$

(6) $$T_{b}=\frac{E}{R}$$

where $n^{*}$ is a power law index; *T* is the static temperature; $\tau^{*\;}$ is the critical shear stress; α is the degree of cure; $\alpha_{g}$ is the degree of cure at gel point; $C_{1}$, $C_{2}\;$ are the fitting constants; $A_{1}$ is the exponential-fitted constant; and $T_{b}$ is the reaction activation energy constant. The values of the Cross–Castro–Macosko model constants in Equation (4) are given in Table 1.

$\frac{d\alpha }{dt}$ is the curing kinetics taken as the Kamal’s model [19]:

(7) $$\frac{d\alpha }{dt}=\left(K_{a}+K_{b}\cdot \alpha^{m}\right)\cdot {\left(1-\alpha \right)}^{n}$$

(8) $$K_{a}=A\cdot \exp\left(\frac{-T_{a}}{T}\right)$$

(9) $$K_{b}=B\cdot \exp\left(\frac{-T_{b}}{T}\right)$$

where $\frac{d\alpha }{dt}$ is the actual cure reaction rate; α is a degree of cure; *m*, *n* are the material constants; $K_{a}$, $K_{b}$ are cure reaction velocity constants; A, B are cure reaction constants; and $T_{a}$, $T_{b}$ are the activation energies. The core values of the Kamal’s model constants in Equation (7) are given in Table 2.

- Volume of Fluid (VOF) [20]

The available free surface of liquid must satisfy two conditions: the free surface kinematic boundary condition (F.S.K.B.C) and the free surface dynamic boundary condition (F.S.D.B.C) [21]. VOF is a method of numerical calculation for establishing the boundary conditions of the basic interface for a free surface of liquid or the interfaces of two fluids. The significant basis of the volume of fluid is to define the fractional function of volume, which, essentially, permits a single element to be filled completely, filled partially, or emptied. Therefore, three regions can be defined through the fractional function of volume, as follows:

(10) $$f=\{\begin{array}{c} 0,\;\mathrm{the}\;\mathrm{element}\;\mathrm{is}\;\mathrm{empty}.\\ 1,\;\mathrm{the}\;\mathrm{element}\;\mathrm{is}\;\mathrm{full}.\\ 0\;<\;f\;<\;1,\;\mathrm{there}\;\mathrm{is}\;a\;\mathrm{fluid}\;\mathrm{interface}\;\mathrm{in}\;\mathrm{element}. \end{array}$$

The fractional volume function is governed by a transport Equation:

(11) $$\frac{\partial f}{\partial t}+\vec{V}\cdot \nabla f=0$$

The following simple serial averages are adopted in this work to approximate the density and viscosity at the interface between fluid 1 and fluid 2.

(12) $$\rho_{f}=f\cdot \rho_{1}+\left(1-f\right)\cdot \rho_{2}$$

(13) $$\eta_{f}=f\cdot \eta_{1}+\left(1-f\right)\cdot \eta_{2}$$

where *f* is the fractional function of volume; $\vec{V}$ is the velocity vector; $\rho_{f}$ is the density of the fractional function of volume; $\rho_{1}$ is the density of fluid 1; $\rho_{2}$ is the density of fluid 2; $\eta_{f}$ is the viscosity of the fractional function of volume; $\eta_{1}$ is the viscosity of fluid 1; $\eta_{2}$ is the viscosity of fluid 2.

### 2.2. Materials

**Figure 1:**
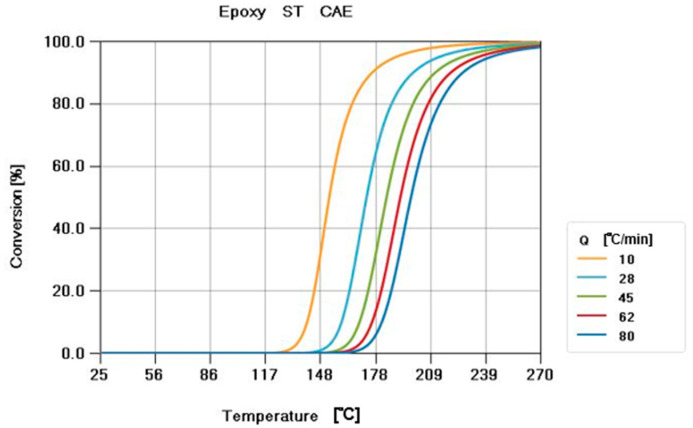
Curing kinetics’ curve of the ST resin. The curing kinetics were measured by a DSC at different temperature ramping rates (Q = 10 °C/min; 28 °C/min; 45 °C/min; 62 °C/min; and 80 °C/min).

Steel putty (ST) resin—the material used in the experiment of motor core gluing in this research—is a thermosetting material. The Metal Industries Research and Development Centre provided ST resin and CoreTech System Co., Ltd, Hsinchu County 30265, Taiwan. measured the material properties. When the temperature rises, the thermosetting epoxy (Perkin Elmer DSC-8500) solidifies, so the curing kinetics should be considered. A thermal DSC measured the curing kinetics (Figure 1). Kamal’s model was used for the curing kinetics model and Kamal’s model Equation is shown in Section 2.1.

**Figure 2:**
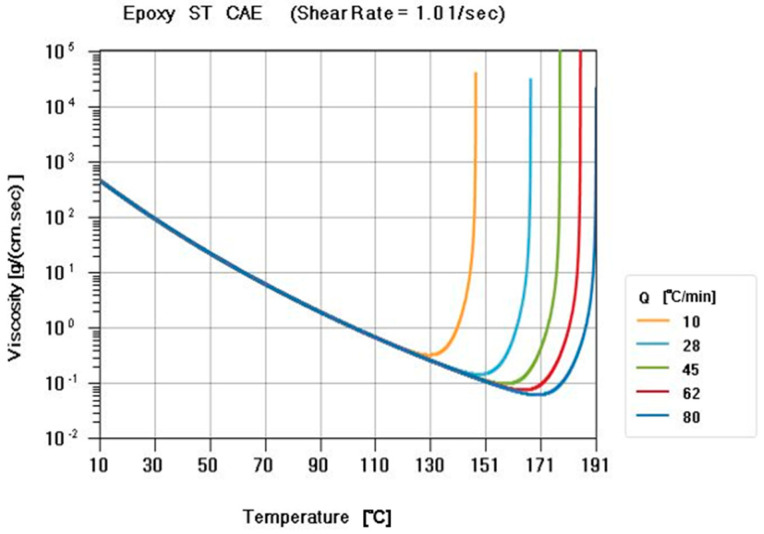
Viscosity curve of the ST resin. The viscosity was measured by a parallel-plate rheometer at different temperature ramping rates (Q = 10 °C/min; 28 °C/min; 45 °C/min; 62 °C/min; and 80 °C/min), and the viscosity changed with time.

A parallel-plate rheometer (Anton Paar MCR502) (Figure 2) provided by CoreTech System Co., Ltd, Hsinchu County 30265, Taiwan was used to measure the defined viscosity of the resin (Perkin Elmer DSC-8500). Because the viscosity always changed in relation to the actual temperature, the curve of viscosity was significant in this experiment. The Cross–Castro–Macosko model was used for the viscosity model, and the Cross–Castro–Macosko model Equation is shown in Section 2.1.

With the above-mentioned theoretical background and measured material properties, the simulation analysis was conducted more reliably and accurately.

## 3. Experiment

### Equipment

**Figure 3:**
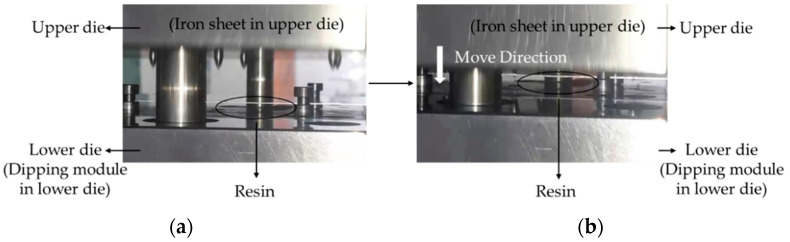
Motor core manufacturing process steps. (**a**) The position of the resin, upper die, and lower die and (**b**) the move direction of the upper die.

Figure 3a shows a motor core formed from iron sheets stacked on each other, which were then glued by the resin between the upper and lower die. Figure 3b shows the move direction of the upper die. The stamping equipment was produced by the Metal Industries Research and Development Centre, Kaohsiung 811225, Taiwan. In this experiment, the resin was moved by a pump, which had a 0.8 MPa driving force; the stamping speed and stamping force were 1.83 mm/sec and 0.022 tf, respectively. The stamping equipment was set to 50 SPM.

**Figure 4:**
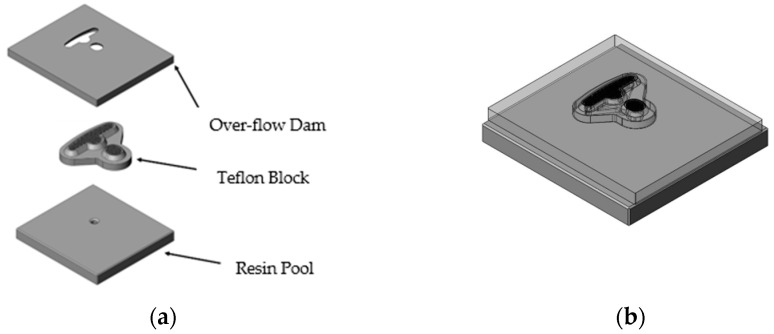
Runner design. (**a**) The individual design of the over-flow dam, the Teflon block, and the resin pool and (**b**) the overall design of the runner.

**Figure 5:**
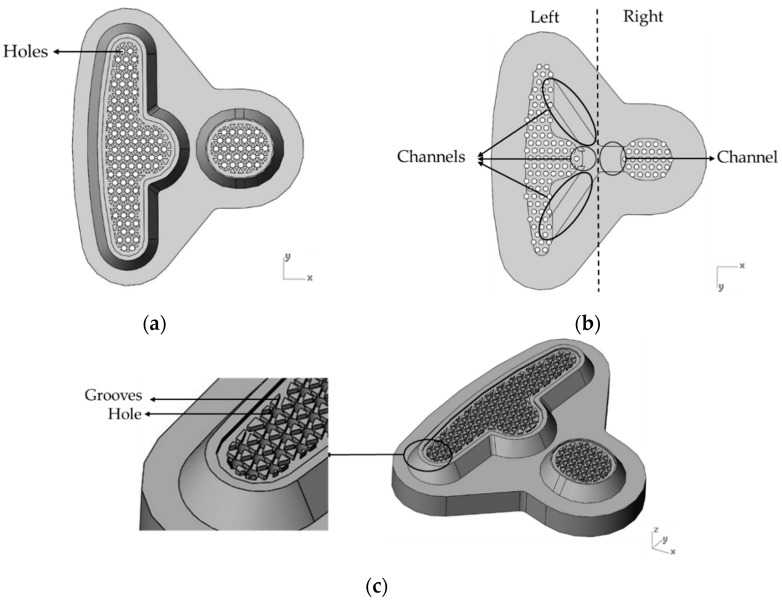
The design of the Teflon module. (**a**) Top view of the Teflon block; (**b**) bottom view of the Teflon block; and (**c**) the microstructure of the Teflon block.

**Figure 6:**
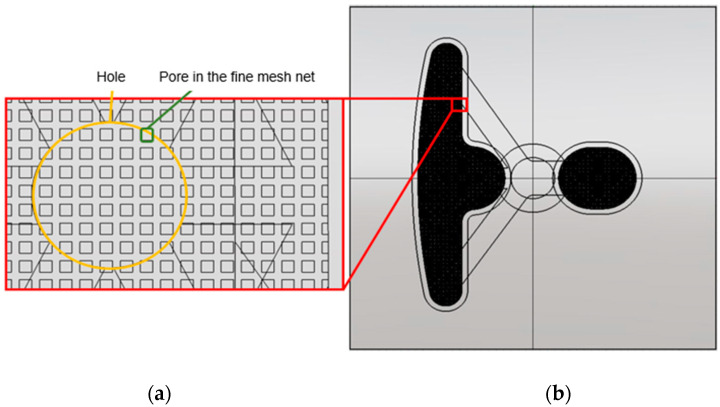
The design of the fine mesh nets. (**a**) Illustration of partial enlargement of fine mesh and (**b**) top view of the design of the fine mesh nets.

Figure 4a shows the individual design of the over-flow dam, the Teflon block, and the resin pool. The runner design is shown in Figure 4b, wherein the design was comprised of three parts: the over-flow dam, Teflon block, and resin pool. The Teflon block design is shown, in detail, in Figure 5. In the top view of the Teflon block, the 122 holes are a Teflon microstructure, where each hole has a 1.5 mm spacing and 1 mm diameter, and are presented in Figure 5a. The internal design of the Teflon block is presented in Figure 5b; it can be seen that the resin flowed into the Teflon block and was divided between the right and left sides by four channels. Figure 5c shows the microstructure on the Teflon block, and the flow of resin was more even due to the design of the grooves. Figure 6a shows an illustration of the partial enlargement of the fine mesh nets, which were used to improve resin distribution. Figure 6b shows the top view of the design of the fine mesh nets.

**Figure 7:**
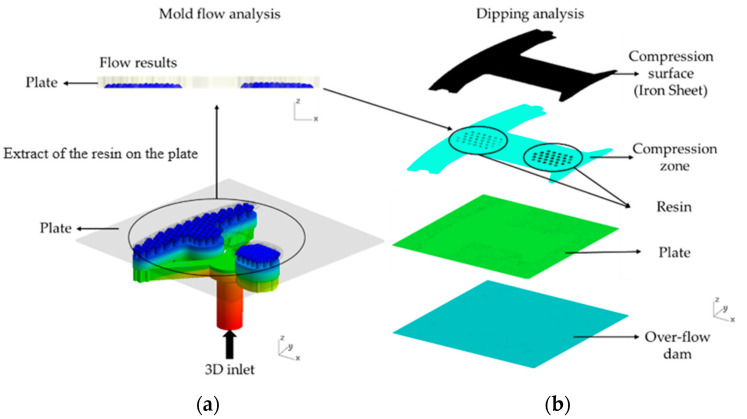
The simulation flow chart. (**a**) The simulation flow chart of the mold flow analysis and (**b**) the simulation flow chart of the dipping analysis.

**Figure 8:**
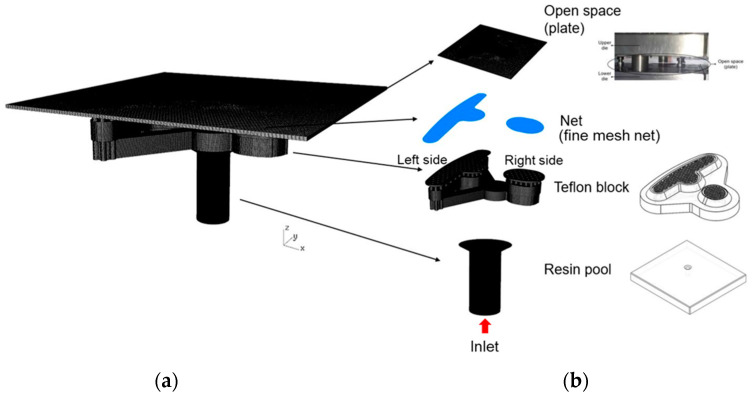
The design of Case 1. (**a**) The overall design of Case 1 and (**b**) the individual design of the plate, the fine mesh nets, the Teflon block, and the resin pool of Case 1.

**Figure 9:**
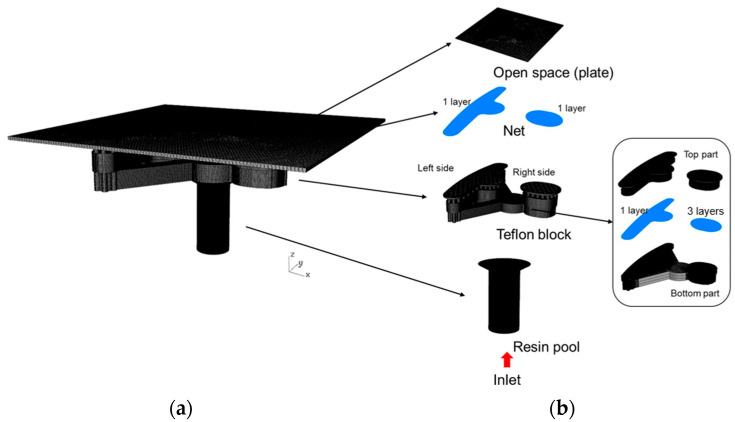
The design of Case 2. (**a**) The overall design of Case 2 and (**b**) the individual design of the plate, the fine mesh nets, the Teflon block, and the resin pool of Case 2.

Figure 7a,b show the simulation flow chart, which includes the mold flow analysis and dipping analysis, where it can be seen that the results of the mold flow analysis were used as the initial conditions for the dipping analysis. In this experiment, there were two different designs of the structures to find out their resin distributions. Figure 8a,b show one design consisted of four parts: the fine mesh nets on the Teflon block, a Teflon block, the over-flow dam, and the resin pool. This design was defined as Case 1. Figure 9a,b show the other design consisted of five parts: the fine mesh nets on the Teflon block, the fine mesh nets between the Teflon block, a Teflon block, the over-flow dam, and the resin pool. This design was defined as Case 2. The purpose of this experiment was basically to explore the impact and influence of fine mesh nets on resin flow and the distribution of the resin on the plate. The fine mesh was evenly distributed on the Teflon block, as can be seen in Figure 6. The pores in the fine mesh nets were squares with a 0.08 mm side length.

By using figures and descriptions of the process and process parameters, in this section, how the actual process was conducted can be clearly conveyed. Therefore, the simulation analysis refers to the above information with regard to the set up.

## 4. Simulation

### 4.1. Mold Flow Analysis Software

Moldex3D 2022 R3PR special edition provided by CoreTech System Co., Ltd, Hsinchu County 30265, Taiwan was used for the analysis in this research. Moldex3D 2020 R3PR special edition is a commercially available computer-aided engineering (CAE) software, which is used mainly for injection molding and compression molding simulations.

### 4.2. Simulation Process

This experiment usually used fine mesh nets to improve the unbalanced state of resin distribution, and the numerical value of the hemisphere-shaped resin in the plate was used as the initial condition for the dipping analysis. Due to the movement of the iron sheet with the upper die during the actual process, the simulation of the compression molding process was used for the dipping process. This research mainly explains and analyzes the entire stacking process, keeping the analysis of the mold flow and the dipping process as the basis for this experiment.

Moldex3D is a powerful simulation tool for the process of injection molding, and it was used to simulate the filling process. In this experiment, this software was used to explore the effect of fine mesh nets on motor core gluing. The fine mesh nets, the resin pool, and the Teflon block constitute the viscous flow channels. In Figure 7, it can be seen that the resin flowed into the module by a pump, and then the resin flowed into the resin pool, the Teflon block, the fine mesh nets, and the plate, sequentially, from the 3D inlet. The hemispheres of resin formed after the resin flowed through 122 holes in the module, and then the hemispheres in the available plate were exported to the compression zone, which was the initial condition of the dipping analysis. Finally, it ended with the gluing of the iron sheets to make a motor core.

### 4.3. Mesh Generation

Rhinoceros 5 provided by CoreTech System Co., Ltd, Hsinchu County 30265, Taiwan was the design software for the mesh modeling that was used in this research. In this research, the mesh models of Case 1 and Case 2 were established for the simulation of the filling process in order to predict the flow trend of the resin, and the mesh model for the dipping process was established to predict the distribution of the resin on the plate.

#### 4.3.1. Filling Process

The Metal Industries Research and Development Centre provided the geometry of the fine mesh nets, the resin pool, and the Teflon block. The diameter of the resin inlet was 6 mm; then, there were three runners that divided the resin to the right and the left side (Figure 5b). Then, the resin flowed through the fine mesh nets, where the actual flow distribution was observed. Finally, the resin flowed out through 122 holes, with a 1 mm diameter, and the resin on the plate was used to observe the flow distribution. Figure 8b shows the Case 1 mesh model, in detail, where there were 8.6 million mesh elements. Figure 9b shows the Case 2 mesh model, in detail, where there were 23.7 million mesh elements. The actual difference between Case 1 and Case 2 was determined after the fine mesh nets were added to the Teflon block.

#### 4.3.2. Dipping Process

**Figure 10:**
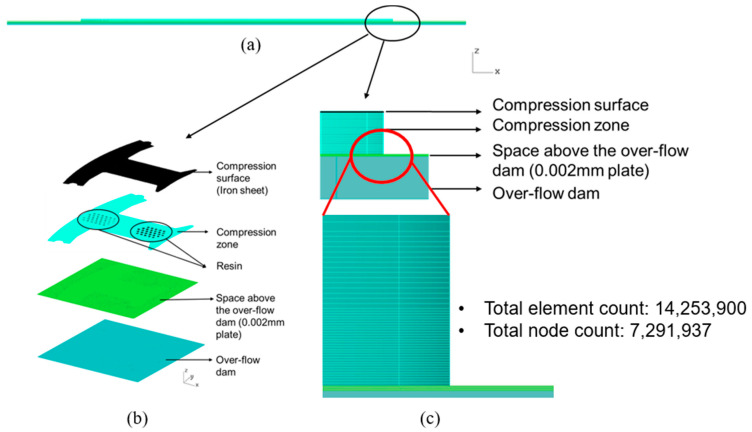
Mesh model used in the dipping process. (**a**) Side view of the overall mesh model used in the dipping process; (**b**) perspective view of the individual mesh model; and (**c**) illustration of partial enlargement of the mesh model used in the dipping process.

The mesh in the impregnation process had four different parts, including the over-flow dam, the space above the over-flow dam (plate), the compression zone, and the compression surface (iron sheet), as shown in Figure 10a–c. The maximum thickness of the resin was 0.002 mm, so the prime simulation was performed by compressing the resin into the space over and above the over-flow dam with a thickness of 0.002 mm, where there were 14.2 million grid elements. In the mesh model of the dipping process, just to save the number of meshes, a gradient mesh was inbuilt inside the compression zone.

### 4.4. Process Parameters

In the simulation, the effect of the resin flow for the filling and dipping processes cannot be ignored. Therefore, the parameters of the experiment were input into the software of mold flow analysis to simulate the flow trend of the resin and predict the distribution of the dipping process.

#### 4.4.1. Filling Process

In the experiment, the resin was pressed, at a pressure of 0.8 MPa, by a pump. Therefore, the actual injection pressure of the simulation setting was 0.8 MPa. For the duration of the setting period, the temperature of the mold and the resin was 45 °C; the resin temperature was about 45 °C, even before entering into the module. The mold temperature of the analysis was set at 45 °C before the resin flowed, isothermally, into the module. This was because the resin was taken out at room temperature (25 °C) for one day, and the initial conversion rate recorded almost no change at all; the initial conversion rate was set to 0% for the analysis. The parameters of the filling process in Case 1 and Case 2 are shown in Table 3 and Table 4. The volume flow rate was 0.00053 cm^3^/s in the experiment. In the simulation analysis, the volume of the mesh model and the filling time to calculate the actual volume flow rate was used. The volume of the mesh model in Case 1 and Case 2 was 4.16 cm^3^, identically. The considerable filling time in Case 1 was set to 138 s, for which the volume flow rate was 0.03 cm^3^/s. The filling time in Case 2 was set to 30 s, for which the volume flow rate was 0.14 cm^3^/s.

#### 4.4.2. Dipping Process

According to the guidelines of the Metal Industries Research and Development Centre, the compression speed is the stamping speed, and the compression force is the stamping force in the stamping process. The focused thickness of the compression zone was 0.5 mm. The real compression gap divided by the compression speed can obtain the true compression time. In accordance with the data provided by the Metal Industry Research and Development Center, the mold temperature and the resin temperature were both set to 45 °C, so that the resin temperature just before entering the module was about 45 °C. Because the resin in the module did not flow over and above 1 h between the actual process of filling and the stage of gluing, the initial conversion rate was set to 0% in Moldex3D mold flow analysis software. The parameters of the dipping process are shown in Table 5.

## 5. Results and Discussion

This section discusses the influence of the flow front of the runner with the fine mesh nets and compares the resin distribution on the plate of the simulation and the actual experiment, and also explains why the measured time difference was used to determine the trend, which is very much similar, rather than the total filling time. This section also discusses the resin distribution during the dipping process, the negative effects due to over-flow, the improvement of the over-flow problem with fine mesh nets, and the error between the simulation results of the dipping process and the target value.

### 5.1. The Influence of Flow Front of The Runner with The Fine Mesh Net

**Figure 11:**
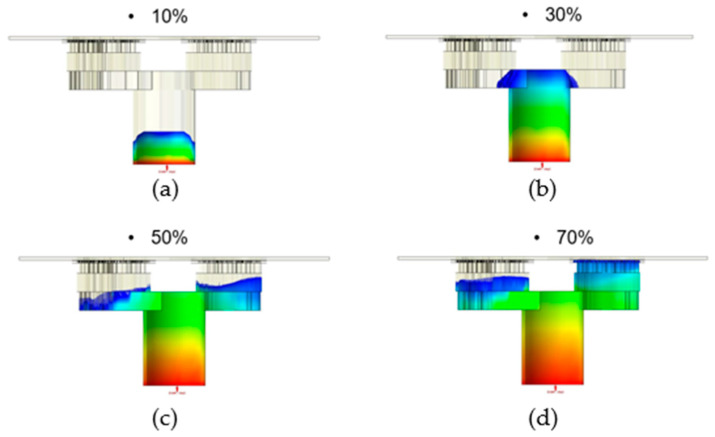
The flow front of the runner with a flow rate of 0.03 cm^3^/sec in Case 1. (**a**) Filling 10%; (**b**) filling 30%; (**c**) filling 50%; and (**d**) filling 70%.

**Figure 12:**
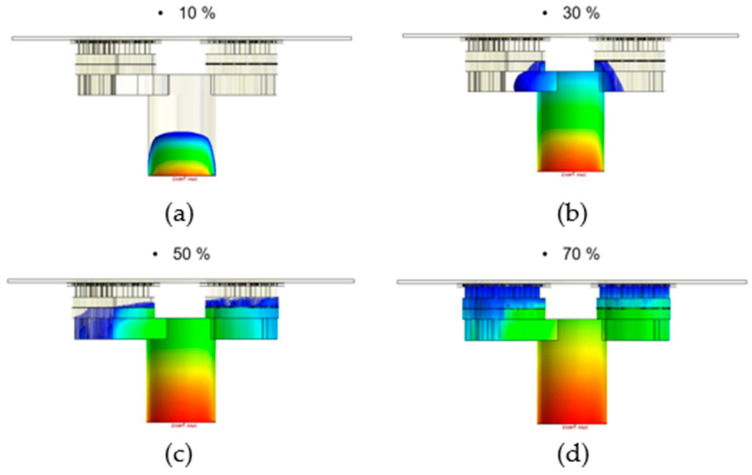
The flow front of the runner with a flow rate of 0.14 cm^3^/sec in Case 2. (**a**) Filling 10%; (**b**) filling 30%; (**c**) filling 50%; and (**d**) filling 70%.

The flow front of the runner on the right side was considerably faster than on the left side, obviously, when the runner was without the fine mesh nets [1]. This situation occurred because the volume on the right side was significantly less than the volume on the left side, so the filling speed on the right side was faster than on the left side. Therefore, the structure of the fine mesh was used to control the filling speed on the right side. The ultimate results of the filling process, which were taken as the initial condition for the dipping process analysis, may have been caused due to the over-flow on the plate; therefore, the flow front of the runner, which was uneven, is in urgent need of improvement. The filling process can also be improved with fine mesh nets; in this research, the fine mesh nets improved the uneven flow front of the runner. In Case 1, the fine mesh net, which consisted of one layer, was applied on the right side and left side of the runner. The flow front of the runner in Case 1 is shown in Figure 11a–d, which show that the flow front of the runner on the right side was considerably faster than on the left side, obviously. The uneven flow front was not improved, obviously. Therefore, the fine mesh net applied to the right side was increased to three layers in Case 2. Figure 12a–d show that the flow front of the runner in Case 2 was more even than in Case 1. Therefore, the set of fine mesh nets, which were in the Teflon block in Case 2, gradually improved the flow front of the runner to further avoid the over-flow problem.

### 5.2. The Resin Distribution on The Plate of Simulation and Experiment

**Figure 13:**
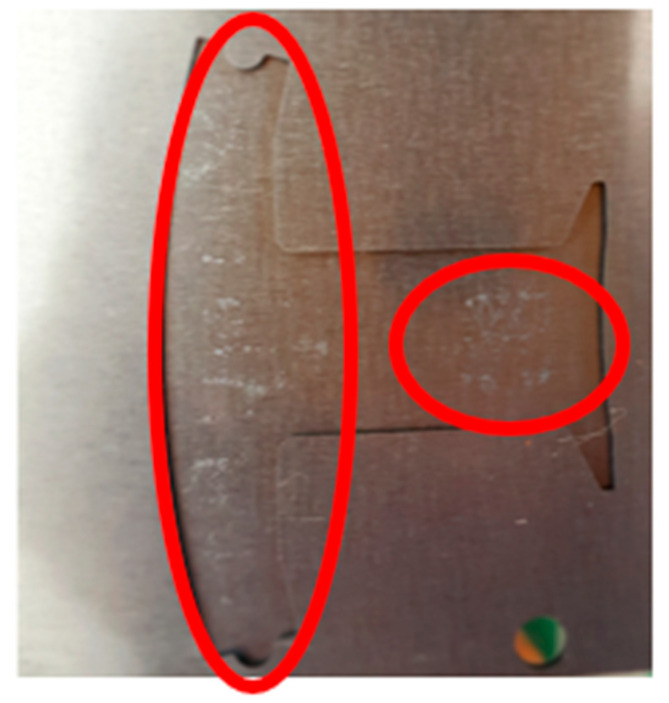
The experimental results of the Case 2 dipping process.

The simulation results of the flow rates of 0.03 cm^3^/sec in Case 1 and 0.14 cm^3^/sec in Case 2, which were calculated by dividing the basic volume of the mesh model by the filling time, are the flow rates that were very much closest to the actual experimental conditions. The simulations of the flow rates were compared with the actual experiment in this research. Since the filling process of the experiment was not so easy to observe, and there was a large difference in the total filling time, the time difference was used to determine whether the trend was similar. The flow front of the runner in Case 2 was more even than in Case 1, which led to differences in the resin distribution on the plate. The experimental and simulation results of the resin flow trend on the plate, for Case 1, are shown in Table 6. The experimental and simulation results showed that the resin distribution on the right side was more than that on the left side, which, ultimately, led to bumps caused by resin over-flow in the dipping process. Table 7 and Figure 13 show the simulation results of the resin flow trend on the plate and the experimental result of the dipping process for Case 2. The final results showed that the flow trend on the plate of Case 2 was more even than in Case 1, so the fine mesh nets in the Teflon block made a significant improvement in the flow trend.

### 5.3. Results of the Dipping Process

**Figure 14:**
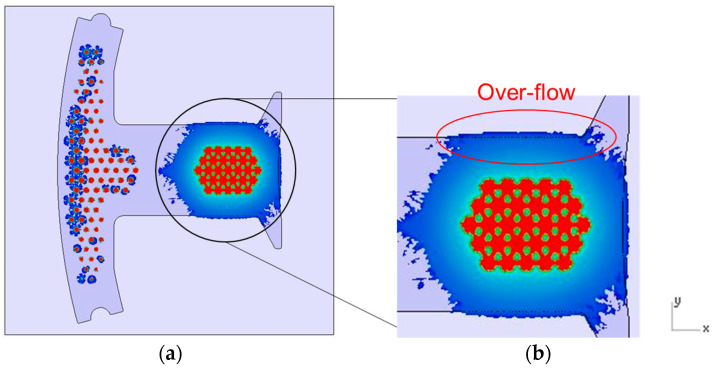
The dipping process results without the fine mesh nets. (**a**) Top view of the dipping process results without the fine mesh nets and (**b**) illustration of partial enlargement of the dipping process results without the fine mesh nets.

**Figure 15:**
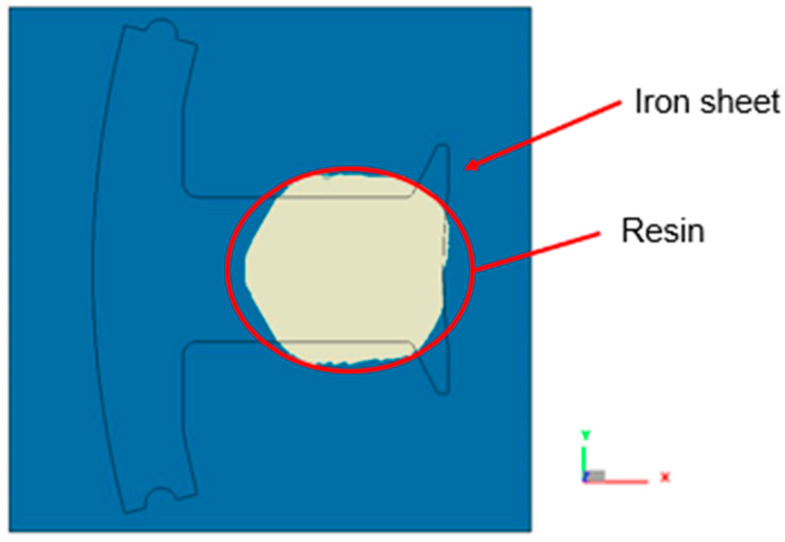
Top view of the dipping process results of Case 1.

**Figure 16:**
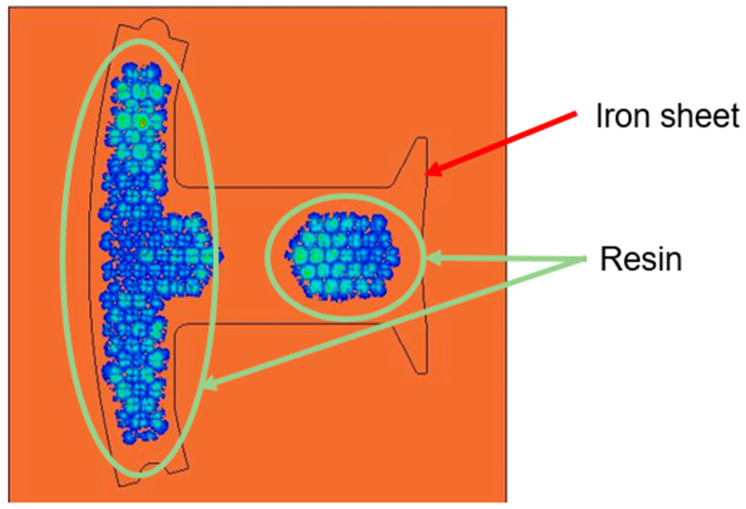
Top view of the dipping process results of Case 2.

**Figure 17:**
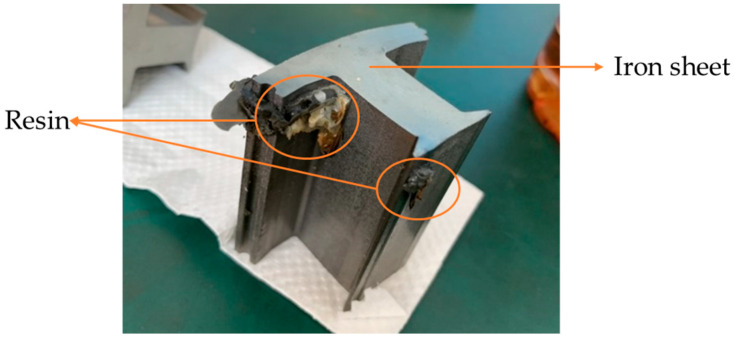
Schematic diagram of bumps caused by resin over-flow.

From the previous research [1], Figure 14a shows the dipping process results without the fine mesh nets, and Figure 14b shows that there is an over-flow problem on the right side, which, ultimately, causes bumps when the iron sheets are stacked on each other (the area of the resin is larger than the size of the iron sheet). In order to improve the bumps, fine mesh nets, in Case 1 and Case 2, were used to improve the over-flow problem. From Table 6, the results of the filling process for Case 1 can be seen and show that the resin emerged first from the right side, then the resin emerged next from the left side. The data of the emerged resin on the plate were extracted for the initial condition of the dipping process analysis. Figure 15 shows the dipping results for Case 1, which actually showed that the over-flow problem did not much improve, as the number of fine mesh nets on the right and left sides were the same. Figure 16 shows that Case 2, with more fine mesh nets, improved the over-flow problem. Because the number of fine mesh nets in Case 2 was increased on the right side of the Teflon block, the dipping results were more even than in Case 1. Figure 13 and Figure 16 show that the simulation results were very much similar to the experimental results in Case 2. Figure 17 shows the bumps caused by the resin over-flow.

## 6. Conclusions

This study focused on the effect of the distribution of a resin filling with fine mesh nets and the technology of the gluing process. In this study, Moldex3D was used to predict the filling and dipping processes in the design of Case 1 and Case 2. First, the simulation of the flow trend of the resin was analyzed, and then the results of the dipping process were predicted. The initial condition for the dipping process was the filling results of the resin on the plate. Finally, the flow of the resin, due to the fine mesh nets, was predicted to improve and, ultimately, resulted in the effect of the fine mesh nets improving the distribution of the resin flow. According to the results of this study, the following conclusions are listed:

1. Because the properties of the plastic in the filling simulation were the most important factors, defining the parameters of the material by Kamal’s reaction dynamic model and the Cross–Castro–Macosko viscosity model allowed the mold flow simulation to analyze more accurately.
2. The results of the distribution of the resin filling showed that the distribution of the resin in Case 2 was more even than in Case 1. The important factor in Case 2 was the addition of more fine mesh net structures in the right side of the Teflon block.
3. The flow front of the runner was more even, due to the fine mesh nets in the Teflon block, and the over-flow problem was further improved. Therefore, the fine mesh nets not only led to the flow trend being more even, but also could avoid bumps.

## Figures and Tables

**Table 1:** Values of the Cross–Castro–Macosko model constants. The constants were measured by a parallel-plate rheometer.

Process Parameters	Parameter Values	Unit
$\alpha_{g}$	0.3	-
$C_{1}$	5.3193	-
$C_{2}$	−10	-
$A_{1}$	$1\times 10^{-9}$	g/(cm·s)
$T_{b}$	7996	K
$n^{*}$	0.8	-
$\tau^{*}$	10	dyne/cm^2^

**Table 2:** Values of the Kamal’s model constants. The constants were measured by a differential scanning calorimeter (DSC).

Process Parameters	Parameter Values	Unit
m	0.75648	-
*n*	2.0614	-
A	228.29	1/s
B	$2.5826\times 10^{7}$	1/s
$T_{a}$	14,586	K
$T_{b}$	8619	K

**Table 3:** The parameters of the filling process in Case 1.

Process Parameters	Parameter Values
Injection pressure (MPa)	0.8
Melt temperature (°C)	45
Mold temperature (°C)	45
Initial conversion (%)	0
Filling time (s)	138

**Table 4:** The parameters of the filling process in Case 2.

Process Parameters	Parameter Values
Injection pressure (MPa)	0.8
Melt temperature (°C)	45
Mold temperature (°C)	45
Initial conversion (%)	0
Filling time (s)	30

**Table 5:** The parameters of the dipping process in Cases 1 and 2.

Process Parameters	Parameter Values
Compression time (s)	0.273
Compression gap (mm)	0.5
Compression speed (mm/sec)	1.83
Compression force (tf)	0.022
Resin temperature (°C)	45
Mold temperature (°C)	45
Initial conversion (%)	0

**Table 6:** Experimental and simulation results of the Case 1 flow trend on the plate.

Experiment	Simulation	Experiment (Δ*t*)	Simulation (Δ*t*)
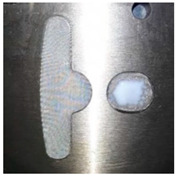 40 s	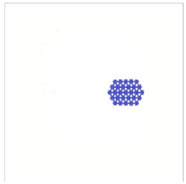 96 s	-	-
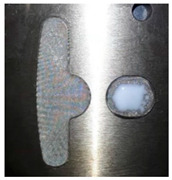 43 s	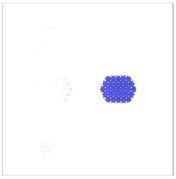 99 s	3 s	3 s
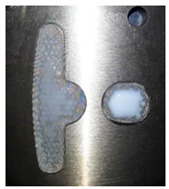 45 s	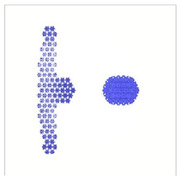 102 s	2 s	3 s

**Table 7:** The simulation results of the Case 2 flow trend on the plate.

Simulation	Filling Percentage
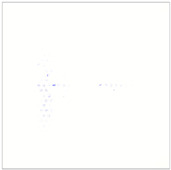	70%
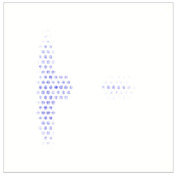	70.5%
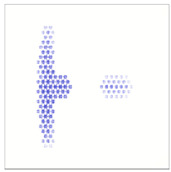	71%
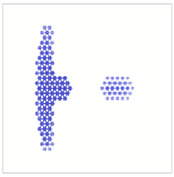	71.5%
